# Alcohol drinking leads to sex-dependent differentiation of T cells

**DOI:** 10.1007/s00068-024-02732-3

**Published:** 2025-01-27

**Authors:** Ramona Sturm, Florian Haag, Christian B. Bergmann, Ingo Marzi, Borna Relja

**Affiliations:** 1 Department of Trauma Surgery and Orthopedics, Goethe University, University Hospital, Frankfurt, Germany; 2 Clinic for Radiology and Nuclear Medicine, Heidelberg University, University Medical Centre Mannheim, Mannheim, Germany; 3 Translational and Experimental Trauma Research, Department of Trauma, Hand, Plastic and Reconstructive Surgery, University Ulm, Albert-Einstein-Allee 23, 89081 Ulm, Germany

**Keywords:** Alcohol consumption, Naïve t cells, Regulatory t cells, Gender, Inflammation

## Abstract

**Objective:**

Global per capita alcohol consumption is increasing, posing significant socioeconomic and medical challenges also due to alcohol-related traumatic injuries but also its biological effects. Trauma as a leading cause of death in young adults, is often associated with an increased risk of complications, such as sepsis and multiple organ failure, due to immunological imbalances. Regulatory T cells play a crucial role in maintaining immune homeostasis by regulating the inflammatory response. Since it is crucial to understand the effects of alcohol in healthy volunteers, in order to refer findings from trauma cohorts, this study investigates the time- and dose-dependent modulation of CD4^+^ lymphocytes and their subsets following acute alcohol consumption, considering both general and sex-specific variations.

**Methods:**

Twelve female and ten male healthy volunteers consumed twelve alcohol mixed drinks over four hours to achieve a blood alcohol level of 1.0‰. Blood samples were collected before and at various time points (2, 4, 6, 24 and 48 h) post-consumption for flow cytometric analyses of the phenotype and activation makers CD4/CD25/CD127 of CD4^+^ T cells and their subtypes.

**Results:**

CD4^+^ lymphocytes significantly decreased at 4 h and increased at 6 h post-alcohol consumption. Naïve CD25^−^CD127^+^ T cells significantly decreased from 2 to 24 h in women and 2 to 48 h in men, while CD25^+^CD127^+^ effector T cells significantly increased during the same period. Natural CD25^+^CD127^−^ regulatory T cells increased significantly at 4 and 6 h, with a higher increase in men. Induced regulatory T cells (CD4^+^CD25^high^CD127^−^) significantly increased at 2 h for all volunteers, with lower proportions of natural and induced regulatory T cells in women.

**Conclusions:**

Acute alcohol consumption induces immune modulation persisting for days, impacting T cell subsets differently in men and women. The prolonged modulation in men may contribute to slightly poorer clinical outcomes, emphasizing the need to consider these effects in trauma patients with acute alcohol intoxication.

## Abstract (plain language summary)

When young adults experience trauma, it is often linked to alcohol use and can lead to serious complications such as infections and organ failure. Our study focused on how the immune system, specifically regulatory T cells, responds to acute alcohol consumption, considering both general trends and differences between healthy men and women. 12 female and 10 male volunteers drank twelve alcoholic beverages over four hours to reach a specific blood alcohol level of 1 per mille. Their blood was obtained at various time points (2, 4, 6, 24, and 48 h) to study the CD4^+^ lymphocytes and their subsets using flow cytometry. The results showed that CD4^+^ lymphocytes initially decreased at 4 h and then increased at 6 h after alcohol consumption. Specific types of T cells had varying responses over time, with differences between men and women. For example, certain regulatory T cells increased more in men at 4 and 6 h. In conclusion, acute alcohol consumption has a lasting impact on the immune system, affecting T cell subsets differently in men and women. This prolonged effect in men may contribute to slightly worse clinical outcomes, highlighting the importance of considering these immune effects in trauma patients with acute alcohol intoxication.

## Highlights

- Trauma, often linked to alcohol use, increases the risk of complications like sepsis and organ failure due to immune imbalances.
- Regulatory T cells, critical for immune balance post-trauma, were studied concerning acute alcohol consumption.
- After drinking, CD4^+^ lymphocytes initially decreased at 4 h and increased at 6 h, showing a dynamic immune response.
- Different T cell subsets had distinct responses over 48 h, with men exhibiting a prolonged immune modulation.
- This extended immune modulation in men could impact clinical outcomes, emphasizing the importance of considering alcohol’s effects in trauma patients.

## Introduction

In recent decades, there has been a significant global increase in *per capita* alcohol consumption, accompanied by a rise in the proportion of heavy episodic drinkers, a trend expected to persist [1]. This poses a substantial socioeconomic and medical challenge. Notably, alcohol consumption is a key factor in the occurrence of injuries [2]. The number of alcohol-related traumata is increasing due to higher alcohol consumption in the population, which raises the likelihood of more people being involved in accidents under the influence of alcohol. Behavioral changes under the influence of alcohol, such as impaired judgment, delayed reaction time, and reduced coordination, lead to a risky behavior and thus increase the risk of accidents. Trauma ranks among the top ten global causes of death, particularly standing as the leading cause of death in young adults [3]. Survivors of severe injuries, including those with acute blood loss, extensive tissue damage, or severe craniocerebral trauma, are known to be at a heightened risk of developing posttraumatic immunological imbalances. These imbalances involve cytokines and cell compositions and may lead to inflammatory complications such as prolonged stays in the Intensive Care Unit (ICU), sepsis, multiorgan failure, and overall poor outcomes [4, 5]. Of particular interest are sex-specific differences, with women exhibiting lower frequencies of complications and experiencing e.g. better outcomes following posttraumatic sepsis [6]. To understand and further investigate the effects of acute alcohol consumption in trauma patients in the context of the complex posttraumatic phase, a comprehensive foundational study of the acute effects of alcohol on healthy subjects is essential. Therefore, in the present study, we examine healthy subjects to assess the pure alcohol-induced effects on the immune system.

Several investigations point to the immunomodulatory impact of alcohol. In alcoholized polytrauma patients, accidents are more frequently attributed to low falls (< 3 m) and penetrating injuries compared to non-alcoholized patients [7]. They demonstrate a significantly elevated likelihood of experiencing an isolated traumatic brain injury, and research indicates a more favorable outcome for alcoholized patients with traumatic brain injuries [8]. In terms of immunosuppressive effects, alcoholized patients exhibit lower leukocyte counts and serum IL-6 levels compared to non-alcoholized trauma patients [9]. Alcoholized patients also manifest a higher incidence of organ failure [10]. Numerous clinical studies have explored the outcome and mortality of alcoholized trauma patients, yielding diverse findings [11–14].

Following the posttraumatic inflammatory response through the non-specific innate immune system, there is a migration of antigen-presenting cells into lymphoid organs, initiating a specific systemic immune response. Depending on the cytokine milieu, naïve CD4^+^ T lymphocytes differentiate into corresponding effector cells (T_H_1, T_H_2, T_H_17, T_FH_) upon antigen contact, activating target cells and eliciting a specific immune response [15]. Post-activation, effector cells express the α-chain of the IL-2 receptor (CD25). Regulatory T cells (Tregs) play a pivotal role in maintaining immune homeostasis and tolerance by suppressing inflammatory processes, eliminating autoreactive T cells, and inducing self-tolerance. Particularly in the post-traumatic phase, Tregs seem crucial for immunological balance [16]. Within Tregs, a distinction exists between natural Tregs (nTregs), originating in the thymus, and induced Tregs (iTregs) [17], which differentiate in the periphery from naïve T cells, likely exerting stronger suppressive effects *via* IL-10 [18]. Tregs modulate both antigen-presenting cells and effector T cells, influencing both innate and adaptive immunity. The differentiation of T cells involves the activation and surface markers of the CD4 receptor on all T cells, as well as the variable expressions of CD25 [19] and the α-chain of the IL-7 receptor (CD127) [20]. In contrast, the effects of alcohol on the adaptive immune system, particularly the regulation and interactions of T lymphocytes post-trauma, are less explored. The limited available results are primarily derived from in vivo research.

The impact of alcohol on the organism, particularly in the context of trauma, is intricate, and discerning the underlying mechanisms responsible for alcohol’s influence on immune responses is challenging. Thus, the current study sought to examine the time- and dose-dependent effects of alcohol on the T cells of healthy individuals, aiming to establish a foundation for understanding the implications for polytraumatized individuals with alcohol involvement.

## Patients AND methods

### Ethics

This study was performed at the University Hospital of the Goethe-University Frankfurt with institutional ethics committee approval (255/14), in accordance with the Declaration of Helsinki in its most recent form and following the Strengthening the Reporting of Observational studies in Epidemiology (STROBE)-guideline [21]. This study is part of a larger study with multiple analyses assessments [22–24]. All healthy volunteers signed the written informed consent form in accordance with ethical standards after detailed explanation of the procedure, effects and objectives of the investigation.

### Study setting and population

Twenty-two healthy participants, comprising ten men and twelve women aged between 18 and 50 years, were included in the study. Those with chronic alcohol consumption, pre-existing immunological disorders, chronic inflammatory diseases, HIV, hepatitis, or ongoing immune-suppressive medication were excluded. Hepatic and renal insufficiencies were ruled out through prior blood examinations. Before the experiment, a comprehensive medical history was taken to exclude chronic diseases. All healthy subjects demonstrated normal kidney function (GFR), with normal creatinine and urea levels, as well as electrolytes, before the experiment commenced. Additionally, liver dysfunctions were excluded through laboratory tests, and GOT, GPT, and GGT levels were measured. Additionally, a detailed alcohol history, including the standardized “Alcohol Use Disorders Identification Test” was conducted to exclude individuals with regular or chronic alcohol consumption.

### Study protocol

Utilizing the modified Widmark equation, an individualized amount of alcohol was calculated, accounting for sex, age, weight, and height, with the aim of achieving a targeted blood alcohol concentration (BAC) of 1‰ one hour after the conclusion of alcohol consumption (four hours). Initially, all participants received a standardized meal, followed by the consumption of a mixed drink comprising Tennessee Whiskey (Jack Daniel’s, Old No 7., Tennessee Whiskey, 40%Vol., Lynchburg, USA) and Coca-Cola Original Taste (The Coca-Cola Company, CocaCola Europacific Partners Deutschland GmbH, Berlin, Deutschland) in a 1:2 ratio every 20 min for a total of 12 drinks over a 4-hour period. Subsequently, a two-hour observation phase ensued. According to the modified Widmark equation and incorporating the parameters sex, age, weight, and height, the women received individual a total quantity for the entire experiment of 145–350 ml Jack Daniel’s (accordingly 290–700 ml Coca-Cola) and men individual a total quantity of 341–550 ml Jack Daniel’s (accordingly 682–1100 ml Coca-Cola). Throughout the 6-hour examination period, each volunteer also consumed one liter of water.

### Blood sampling

The initial blood sample, designated as the control (T0), was collected prior to the onset of alcohol consumption using lithium-heparin tubes (S-Monovette LH, Sarstedt, Nümbrecht, Germany). Subsequent blood samples were obtained at intervals of 2, 4, 6, 24, and 48 h, following the previously outlined procedures [22–24]. Immediate processing of collected blood was conducted at room temperature. Additionally, the blood alcohol concentration was assessed using a serum-gel tube (S-Monovette, Sarstedt, Nümbrecht, Germany) by the clinical laboratory of the University Hospital Frankfurt.

### Measurement of cell surface receptor expression by flow cytometry

Blood samples (100 𝜇l) were transferred to polystyrene round-bottom tubes (BD Bioscience, Franklin Lakes, NJ, USA) and incubated with specific mouse anti-human CD4 APC/Cy7 (Clone RPA-T4, BioLegend, San Diego, CA, USA), mouse anti-human CD25 APC (Clone BC96, BioLegend, San Diego, CA, USA), and mouse anti-human CD127 FITC (Clone A019D5, BioLegend, San Diego, CA, USA) antibodies. Following a 10-minute incubation at room temperature in the dark, the samples were washed with 2 ml of phosphate-buffered saline (PBS, Gibco, Thermo Fisher Scientific, Waltham, MA USA) supplemented with 0.5% bovine serum albumin (Sigma-Aldrich, St. Louis, USA) (FACS buffer). After removing the supernatants, 3 ml of erythrocyte-lysing solution (PBS + 0.83% NH_4_Cl + 0.1% KHCO_3_ + 0.004% EDTA; Sigma-Aldrich, St. Louis, USA) were added for a 10-minute incubation in the dark on ice. Subsequently, the samples were centrifuged at 1500 rpm for 5 min and were washed with 2 ml FACS buffer. After removing the supernatants, 5 µl 7-Aminoactinomycin D dye (7 AAD Cell Viability; BD Bioscience, Franklin Lakes, NJ, USA) were added for additional 10 min in the dark on ice. The samples were then washed with 2 ml FACS buffer and centrifuged at 1500 rpm for 5 min. Immediately after removing the supernatants, the cells were diluted in 500 𝜇l FACS buffer and measured using a commercial flow cytometric analysis system (FACS Canto 2; BD Bioscience, Franklin Lakes, NJ, USA) and FACS DIVA software (BD Bioscience, Franklin Lakes, NJ, USA). Singlets and cell viability were controlled, and leukocytes and lymphocytes were gated based on their forward- and side-scatter profiles. Subpopulations of CD4^+^ T cells were identified by their distinct expression of phenotype markers CD25 and CD127.

### Statistical analysis

GraphPad Prism 6.0 software (GraphPad Software Inc. San Diego, CA, USA) was used to perform the statistical analysis. Data are given as mean ± standard error of the mean. Kolmogorov-Smirnov test was used to assess the normality of data. The Mann-Whitney U test is performed for statistical comparisons between the sexes. The Wilcoxon test is used for the matched pair analysis of the healthy volunteers over the time course. A p value below 0.05 was considered statistically significant.

## Results

### Study population

Twelve female and ten male healthy volunteers constituted the participant pool for this study. The study population showed a mean age of 25 ± 4 years. One male healthy volunteer did not drink the last two of the 12 mixed drinks, otherwise there were no drop-outs and all healthy volunteers consumed their individually calculated amount of mixed drinks.

### Blood alcohol concentration

At the control timepoint (T0), all healthy volunteers displayed no measurable blood alcohol concentration. By T2, the BAC significantly increased to 0.46±0.02‰ compared to T0 (*p* < 0.05). After four hours, all volunteers, irrespective of sex, achieved the targeted value with a BAC of 1.11±0.05 per mile. By T6, two hours after the end of alcohol consumption, the BAC decreased to 0.83 ± 0.06‰ still significantly elevated compared to T0 (*p* < 0.05) [22]. No detectable blood alcohol concentration was observed after 24 h and 48 h.

**Fig. 1:**
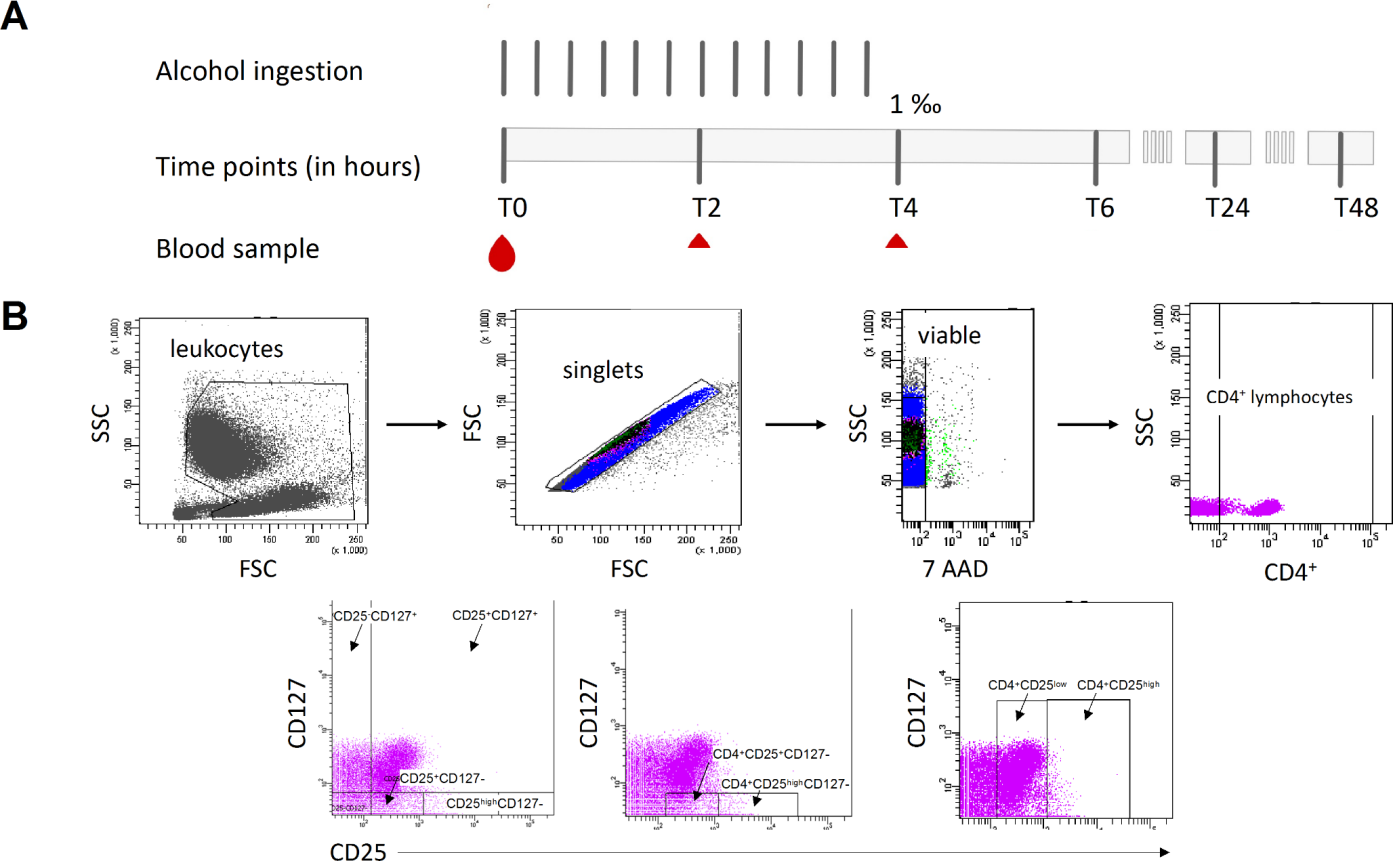
Study design and the gating strategy. **(A)** Twenty-two healthy volunteers (HV) received a standardized lunch one hour before the experiment and one liter water at hand during the first six hours of experiment (T0-T6). The study cohort received an individually calculated amount of Whisky-cola mixture for four hours, reaching blood alcohol concentration of 1‰ after 4 h (T4). Blood samples were collected before (T0) and 2 h (T2), 4 h (T4), 6 h (T6), 24 h (T24) and 48 h (T48) after starting the alcohol drinking. This is the same study design as published before [22–24]. **(B)** Representative gating strategy for the flow cytometric analyses and evaluation of T cells

Sex-specific differences in blood alcohol concentration were not significant throughout the entire study period [22]. The study design and the gating strategy are illustrated in Fig. 1.

### Proportion of CD4+ lymphocytes of all lymphocytes

**Fig. 2:**
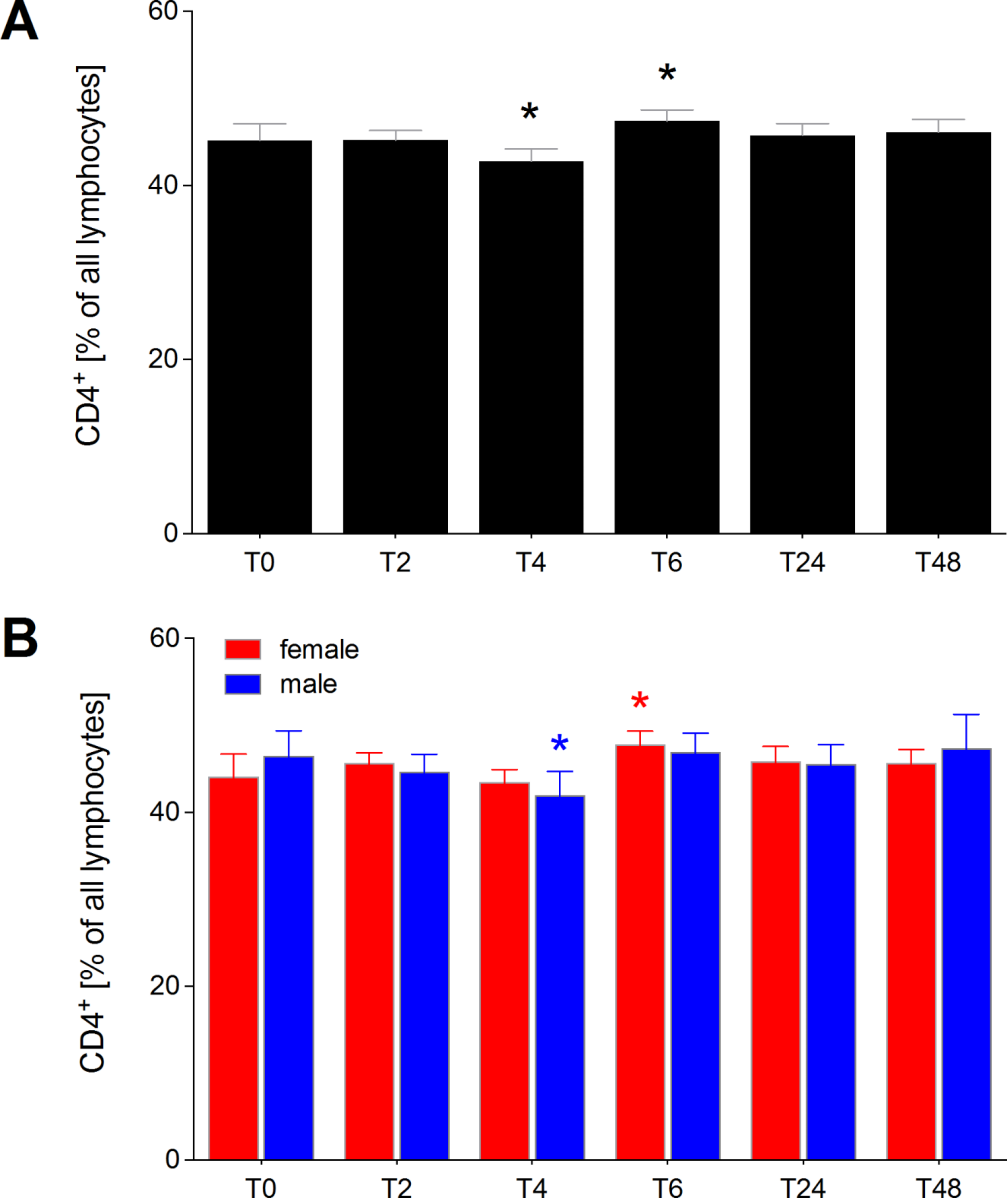
Percentage of CD4^+^ lymphocytes among all lymphocytes in healthy volunteers before, during and after alcohol consumption. CD4^+^ lymphocytes as proportions of all lymphocytes in healthy volunteers before alcohol consumption (ctrl, T0), two (T2), four (T4), six (T6), 24 (T24) and 48 (T48) hours after starting the four-hour drinking period in **(A)** all healthy volunteers (*n* = 22), and **(B)** in female (*n* = 12) versus male (*n* = 10). *(black): *p* < 0.05 vs. indicated, *(red): *p* < 0.05 in female vs. T0 female, *blue: *p* < 0.05 in male vs. T0 male

Four hours after initiation of acute alcohol consumption, there was a significant reduction in the proportion of CD4^+^ lymphocytes in all healthy volunteers (*p* < 0.05, Fig. 2). However, by T6, the proportion significantly increased compared to normal values (*p* < 0.05, Fig. 2A). When analyzed by sex, only women exhibited a significant decrease in the proportion of CD4^+^ lymphocytes (*p* < 0.05. Figure 2B).

Six hours after the onset of alcohol consumption, the proportion of CD4^+^ lymphocytes significantly increased in all healthy volunteers compared to normal values, and this increase was also observed in males (*p* < 0.05, Fig. 2B). At T24 and T48, the distribution of CD4^+^ lymphocytes returned to normal levels for all healthy volunteers, as well as when analyzed separately for males and females (Fig. 2A and B).

### Proportion of CD25−CD127+ naïve T cells of CD4+ lymphocytes

**Fig. 3:**
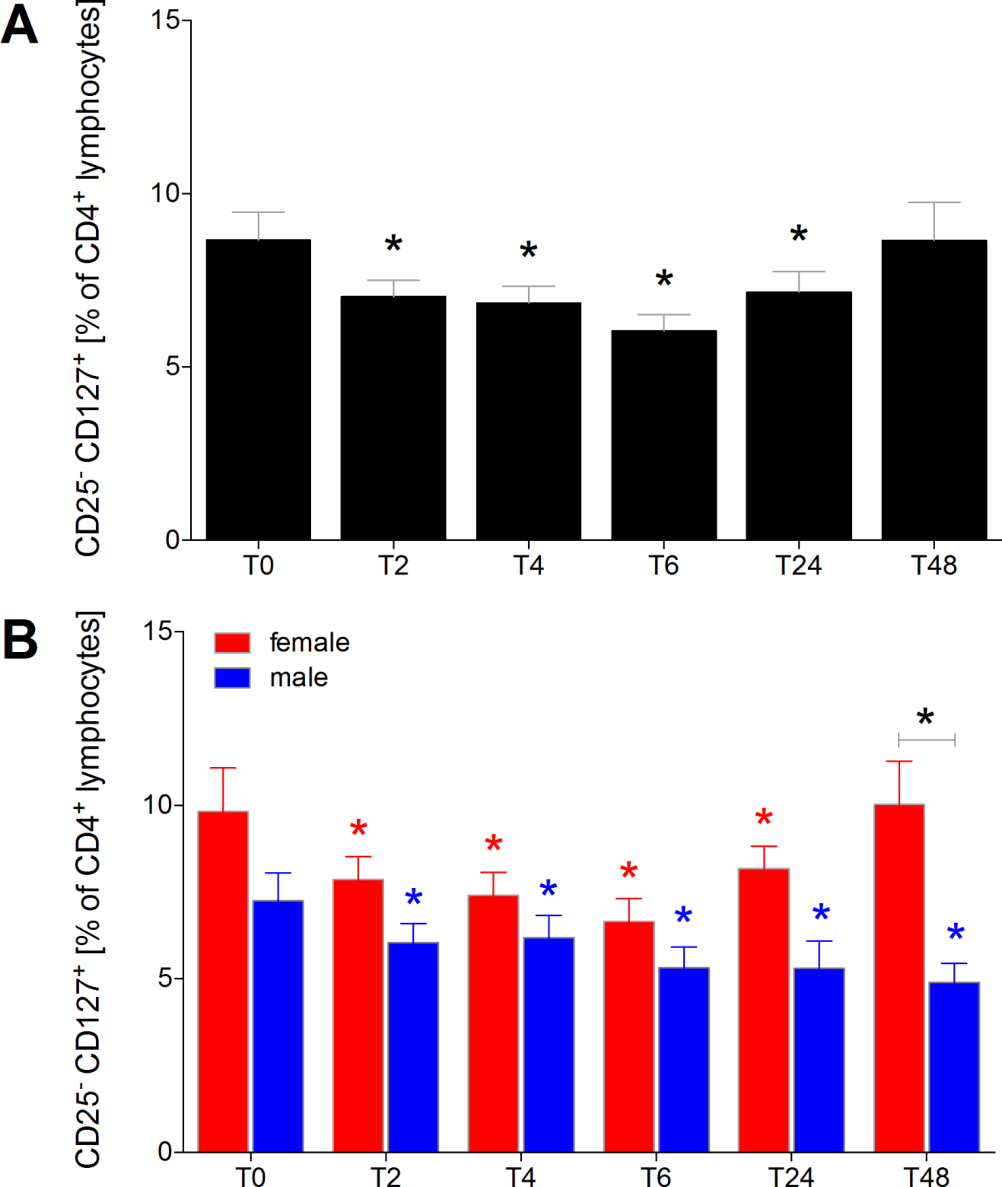
Percentage of CD25^−^CD127^+^ naïve T cells among CD4^+^ lymphocytes in healthy volunteers before, during and after alcohol consumption. CD25^−^CD127^+^ naïve T cells as proportions of CD4^+^ lymphocytes in healthy volunteers before alcohol consumption (ctrl, T0), two (T2), four (T4), six (T6), 24 (T24) and 48 (T48) hours after starting the four-hour drinking period in **(A)** all healthy volunteers (*n* = 22), and **(B)** in female (*n* = 12) versus male (*n* = 10). *(black): *p* < 0.05 vs. indicated, *(red): *p* < 0.05 in female vs. T0 female, *blue: *p* < 0.05 in male vs. T0 male

The proportion of CD25^−^CD127^+^ naïve t cells exhibited a significant decrease at T2 through T24 compared to T0 (*p* < 0.05, Fig. 3A). This reduction remained significant until T48 for both female and male HV compared to T0 (*p* < 0.05, Fig. 3B).

While male HV consistently had significantly lower proportions of CD25^−^CD127^+^ naïve T cells throughout the entire observational period compared to T0, this decrease persisted in females up to T48 (*p* < 0.05, Fig. 3B). Notably, the difference in proportions of CD25^−^CD127^+^ naïve T cells between males and females reached significance at T48 (*p* < 0.05, Fig. 3B). Specifically, the proportion of CD25^−^CD127^+^ naïve T cells among all CD4^+^ lymphocytes was significantly reduced in females compared to males at T48 (*p* < 0.05, Fig. 3B).

### Proportion of CD25+CD127+ T cells of CD4+ lymphocytes

**Fig. 4:**
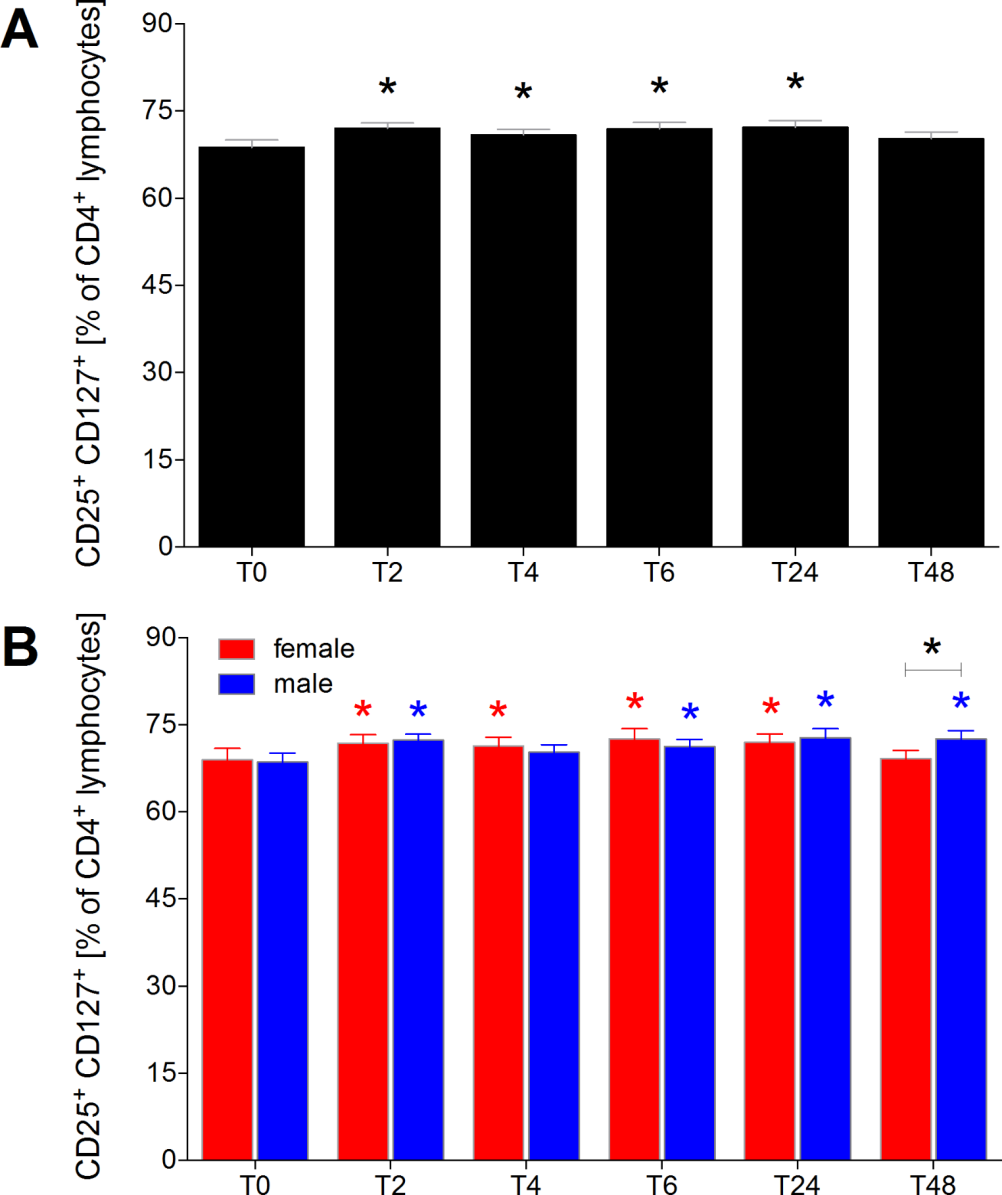
Percentage of CD25^+^CD127^+^ effector T cells among CD4^+^ lymphocytes in healthy volunteers before, during and after alcohol consumption. CD25^+^CD127^+^ effector T cells as proportions of CD4^+^ lymphocytes in healthy volunteers before alcohol consumption (ctrl, T0), two (T2), four (T4), six (T6), 24 (T24) and 48 (T48) hours after starting the four-hour drinking period in **(A)** all healthy volunteers (*n* = 22), and **(B)** in female (*n* = 12) versus male (*n* = 10). *(black): *p* < 0.05 vs. indicated, *(red): *p* < 0.05 in female vs. T0 female, *blue: *p* < 0.05 in male vs. T0 male

Two hours after initiation of alcohol consumption, the proportion of CD25^+^CD127^+^ effector T cells significantly increased and remained significantly elevated in all healthy volunteers until T24 (*p* < 0.05, Fig. 4A). When analyzed by sexes, the proportion of CD25^+^CD127^+^ effector T cells among CD4^+^ lymphocytes in females was significantly elevated from T2 to T24 (Fig. 4B, *p* < 0.05). In males, the proportion of CD25^+^CD127^+^ effector T cells was also significantly increased at T48, leading to a significant difference between the sexes at T48 (Fig. 4B, *p* < 0.05).

### Proportion of CD25+CD127− natural regulatory T cells of CD4+ lymphocytes

**Fig. 5:**
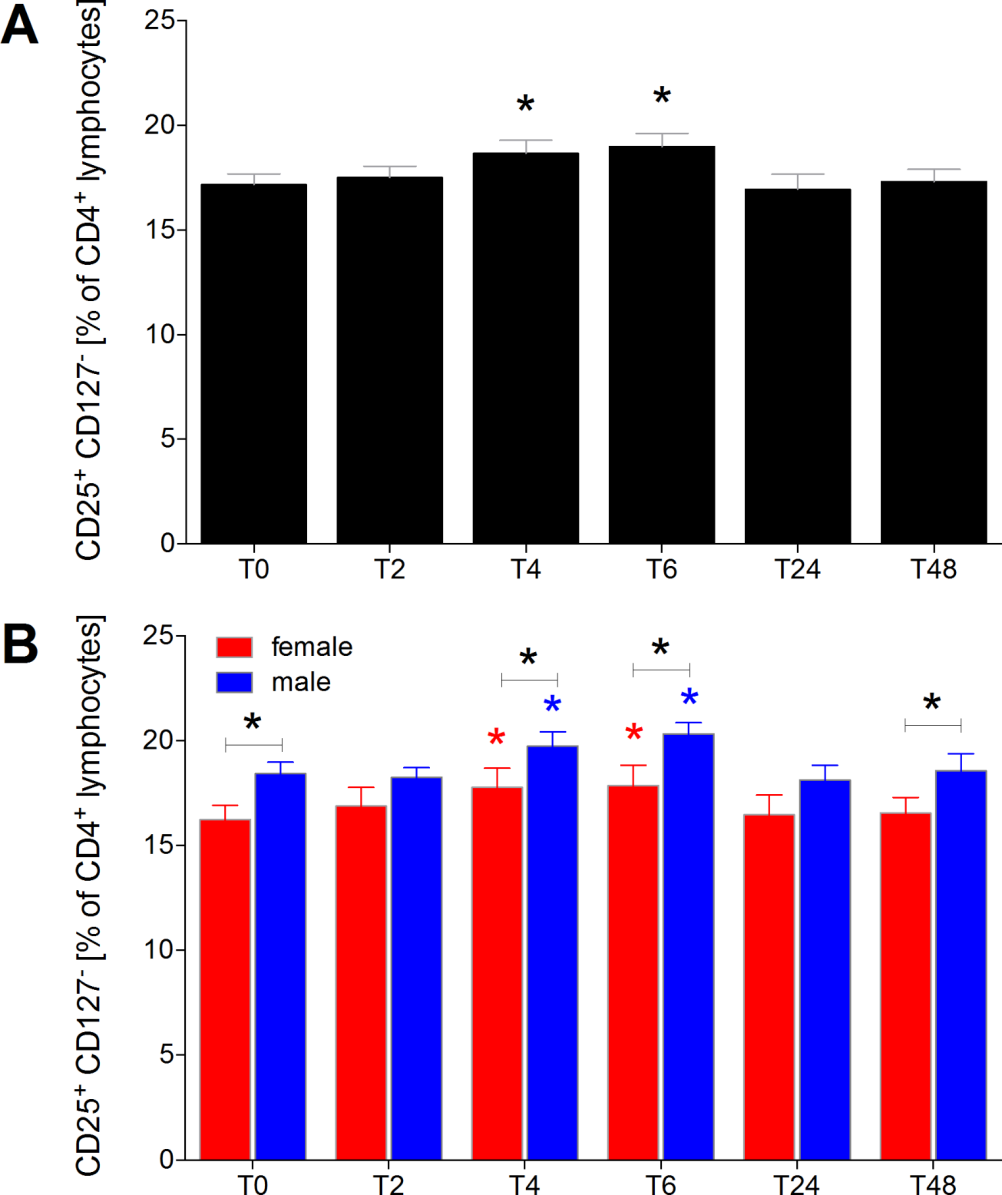
Percentage of CD25^+^CD127^−^ regulatory T cells among CD4^+^ lymphocytes in healthy volunteers before, during and after alcohol consumption. CD25^+^CD127^−^ regulatory T cells as proportions of CD4^+^ lymphocytes in healthy volunteers before alcohol consumption (ctrl, T0), two (T2), four (T4), six (T6), 24 (T24) and 48 (T48) hours after starting the four-hour drinking period in **(A)** all healthy volunteers (*n* = 22), and **(B)** in female (*n* = 12) versus male (*n* = 10). *(black): *p* < 0.05 vs. indicated, *(red): *p* < 0.05 in female vs. T0 female, *blue: *p* < 0.05 in male vs. T0 male

The proportion of CD25^+^CD127^−^ natural regulatory T cells among CD4^+^ lymphocytes was significantly increased at T4 and T6 compared to T0 in HV (*p* < 0.05, Fig. 5A). This increase was significant at T4 and T6 in both female and male healthy volunteers compared to their respective T0 control (*p* < 0.05, Fig. 5B). Under normal conditions at T0 and throughout the entire observational period, female healthy volunteers consistently exhibited lower proportions of CD25^+^CD127^−^ natural regulatory T cells among CD4^+^ lymphocytes compared to males. However, this difference reached significance at T0, T4, T6, and T48 (*p* < 0.05, Fig. 5B).

### Proportion of CD25highCD127− induced regulatory t cells of CD4+ lymphocytes

**Fig. 6:**
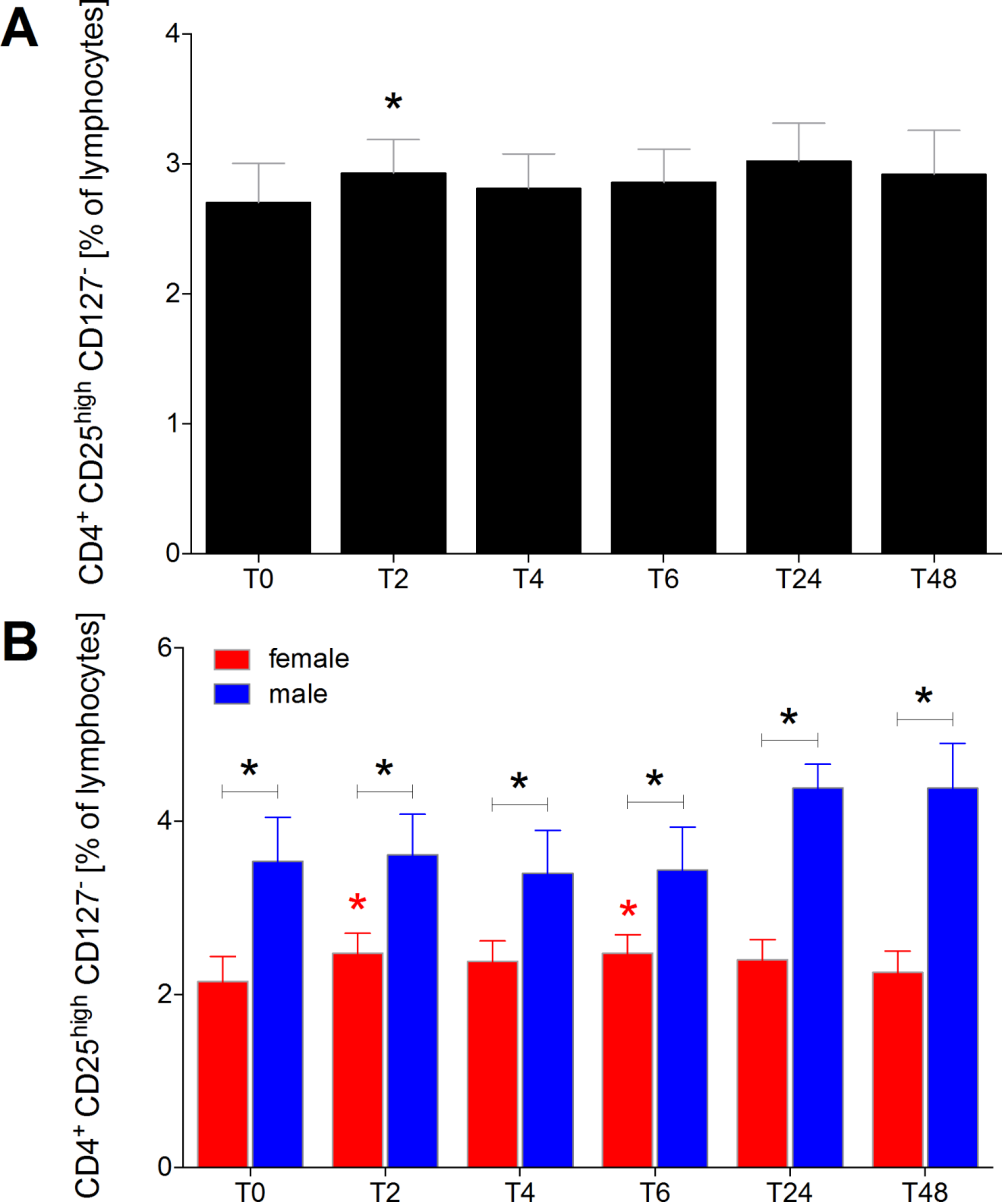
Percentage of CD4^+^CD25^high^CD127^−^ regulatory T cells among CD4^+^ lymphocytes in healthy volunteers before, during and after alcohol consumption. CD25^high^CD127^−^ real regulatory T cells as proportions of CD4^+^ lymphocytes in healthy volunteers before alcohol consumption (ctrl, T0), two (T2), four (T4), six (T6), 24 (T24) and 48 (T48) hours after starting the four-hour drinking period in **(A)** all healthy volunteers (*n* = 22), and **(B)** in female (*n* = 12) versus male (*n* = 10). *(black): *p* < 0.05 vs. indicated, *(red): *p* < 0.05 in female vs. T0 female, *blue: *p* < 0.05 in male vs. T0 male

The proportion of CD25^high^CD127^−^ induced regulatory T cells among CD4^+^ lymphocytes significantly increased two hours after the initiation of alcohol consumption at T2 compared to T0 (*p* < 0.05, Fig. 6A). This increase was significant at both T2 and T6 for female healthy volunteers compared to T0 (*p* < 0.05, Fig. 6B). Throughout the entire observational period, female healthy volunteers consistently exhibited significantly lower proportions of CD25^high^CD127^−^ adaptive regulatory T cells among CD4^+^ lymphocytes compared to males (*p* < 0.05, Fig. 6B).

## Discussion

Polytrauma remains a leading cause of death in young adults globally, with a significant portion of patients being alcohol-intoxicated. Depending on the study, up to nearly 50% of patients may have a positive blood alcohol level [25]. Acute alcohol intoxication further induces immunomodulations, impacting the clinical course (9–10). While data on the effects of alcohol abuse on the primary innate immune response exists, the T cell-mediated specific immune responses, especially the immunosuppressive role of regulatory T cells, are crucial for establishing immunological balance and preventing complications such as sepsis in the posttraumatic phase. In our own studies involving polytraumatized patients with an Injury Severity Score (ISS) of ≥ 16 and blood examinations upon admission to the emergency department, we observed an acute posttraumatic increase in absolute leukocyte counts that subsequently decreased until day six [26, 27]. Additionally, the proportion of human regulatory T cells (Tregs) in the peripheral blood of traumatized patients upon admission to the emergency department was lower compared to healthy volunteers [27].

Human *ex vivo in vitro* studies on the acute alcohol-induced modulation of CD4^+^ T helper cells are currently lacking. Therefore, the present study aimed to investigate the time-dependent effects of acute alcohol on the acquired immune system, specifically focusing on the differentiation of naïve CD4^+^ T cells and the impact on regulatory T cells. Chronic alcohol consumption is known to reduce the number of CD4^+^ T cells, as demonstrated by Boyadjieva et al. in male rats fed an alcoholic liquid diet for four weeks [28]. Additionally, Gheorghiu et al. found that chronic alcoholic abuse decreased the CD4^+^/CD8^+^ cell ratio [29]. In the current study, the proportion of CD4^+^CD25^−^CD127^+^ naïve T cells was significantly decreased from T2 to T24 compared to T0 in the study cohort, suggesting that acute alcohol consumption may reduce the proportion of naïve T cells, persisting even one day after alcohol use. This decrease was significant in both female and male healthy volunteers, with the reduced proportion in men persisting for up to two days after acute alcohol use. Similarly, the proportion of CD4^+^CD25^+^CD127^+^ effector T cells significantly increased as early as two hours after the initiation of alcohol intake, persisting up to 24 h and even the second day after acute alcohol use. These results suggest that alcohol consumption may promote the differentiation from naïve T cells to effector T cells, with detectable changes in both sexes as early as 24 h after drinking, even when blood alcohol concentrations are negative. In men, these differences persist for up to 48 h after acute alcohol consumption. While mechanistic studies on potential naïve T cell activation are lacking, the results indicate that the reduction of naïve T cells may limit the host’s response to damage- and/or pathogen-associated molecular patterns released immediately after trauma, resulting in increased susceptibility to posttraumatic inflammatory complications [30, 31]. McTernan et al. conducted an in vitro investigation on the immune metabolism and differentiation of CD4^+^ cells by plating human naïve CD4^+^ T cells and allowing them to differentiate under Th1-promoting conditions in the presence of IL-12 and ethanol for three days. Their findings demonstrated a differentiation of T cells towards proinflammatory Th1 cells and an inhibition of regulatory T cells. However, the experiment was conducted under limited conditions, involving isolated naïve T cells and targeted stimulation towards Th1 cells [32].

We demonstrated a significant increase in the proportion of CD4^+^CD25^+^CD127^−^ natural regulatory T cells among CD4^+^ lymphocytes four and six hours after alcohol use in both females and males. Interestingly, under normal conditions and throughout the entire observational period, female healthy volunteers consistently had lower proportions of CD4^+^CD25^+^CD127^−^ natural regulatory T cells compared to males. The notably increased proportion of natural regulatory T cells in males may contribute to the better outcomes observed in polytraumatized women compared to men. On the other hand, the proportion of CD4^+^CD25^high^CD127^−^ induced regulatory T cells among CD4^+^ lymphocytes significantly increased two hours after the initiation of alcohol consumption in both sexes. In females, this increase remained significant at both two and six hours compared to controls. However, throughout the complete observational period, female healthy volunteers consistently exhibited significantly lower proportions of CD4^+^CD25^high^CD127^−^ induced regulatory T cells compared to males, and this sex-specific difference was present at baseline levels. Afshan et al. also reported a sex-specific distribution of regulatory T cells in flow cytometric analysis of young adult blood, with higher proportions of regulatory T cells in males [33]. Several studies on trauma cohorts, including those with alcohol-positive patients, have demonstrated worse outcomes regarding head injuries, Glasgow Coma Scale (GCS) scores, and some physiological parameters. However, neither the 24-hour mortality nor the overall mortality showed significant differences between groups. Subgroup analysis in traumatic brain injury (TBI) patients with negative blood alcohol concentrations revealed significantly higher mortality rates compared to those with positive blood alcohol levels. The results remain controversial in the literature, and the mechanisms are not fully elucidated [8]. Importantly, many studies, including those involving alcohol-intoxicated trauma patients, do not stratify between acute and chronic alcohol ingestion. Additionally, there is a lack of data on whether patients were regularly discharged, discharged against medical advice, or underwent inpatient alcohol admission to enhance compliance and prevent alcohol withdrawal, which is common practice in hospitals.

The experimental design mimicked a typical scenario with food and several drinks consumed over the course of the evening, aiming to investigate the immunomodulatory effects at a blood alcohol concentration of one per mille. This represents a limitation of the study, as blood samples were taken at various time points, but alcohol consumption continued. Consequently, no conclusions can be drawn about specific immunomodulation referred to different blood alcohol levels. Further studies with varying amounts of alcohol and subgroup analyses would be necessary to address this aspect.

The present work aims to acquire a foundational understanding of the effects of acute alcohol consumption on T cells and their subsets, to be able to understand the posttraumatic immunomodulation of alcoholized patients in the course of further studies. In a study of polytrauma patients upon admission to the emergency department and over the subsequent 10-days, we demonstrated that the frequency of CD4^+^CD25^high^ and CD4^+^CD25^+^CD127^−^ regulatory T cells was significantly decreased immediately after admission to the emergency department and in the following 10 days post-injury. Compared to healthy volunteers, the proliferation of CD4^+^ T cells in trauma patients increased significantly upon admission and during the subsequent days. As expected, CD4^+^CD25^+^CD127^−^ regulatory T cells reduced CD4^+^ cell proliferation in healthy volunteers; however, in traumatized patients, regulatory T cells seemed to enhanced the CD4^+^ proliferation [34].

In previous studies, we demonstrated that both leukocyte counts and systemic IL-6 levels were significantly reduced upon admission in acutely intoxicated polytrauma patients [9]. In our current study, we observed a decrease in the proportion of naive CD4^+^CD25^−^CD127^+^ lymphocytes. Conversely, there was an increased proportion of effector T cells over 24 h in females and over 48 h in males, as well as an increased proportion of natural and induced regulatory T cells.

In terms of polytrauma, it can be assumed that the immediate posttraumatic pro-inflammatory immunomodulation encounters a pre-primed anti-inflammatory immune state due to alcohol, which modulates the immune response accordingly. The alcohol-induced prolonged changes in T cell subsets, with a prolonged effect in men, may contribute to slightly worse clinical outcome rates in male.

Our findings demonstrate that acute alcohol consumption induces immunomodulations, potentially leading to the differentiation of T cells with an increase in effector T cells and regulatory T cells. The presence of sex-specific differences at baseline levels, as well as persisting up to 48 h after alcohol abuse, emphasizes the importance of considering these factors in the treatment of alcohol-intoxicated trauma patients. These immunological changes may contribute to an imbalance that promotes posttraumatic complications. Thus, the study contributes valuable information to the intersection of trauma, alcohol consumption, and immunology, with potential implications for clinical practice, trauma management, and public health interventions.

## Data Availability

The raw data supporting the conclusions of this article will be made available by the corresponding author upon a reasonable request, without undue reservation.
